# Some Uses of Antimony

**Published:** 1892-03-19

**Authors:** 


### SOME USES OF ANTIMONY.

Antimony is one of those drugs which have experienced extraordinary changes of popularity. At one time the universal remedy, together with bleeding, for half the complaints of life, and then gradually neglected as a dangerous depressant till it has become a perfectly unknown remedy to many practitioners. The unreasoning disuse of bleeding which followed the Brownian controversy was accompanied by equally unreasoning use of stimulants and rich food, and we now combat in vain both the popular reliance which is placed on stimulants and the horror of venesection felt by the non-medical world. Were it not for the popular sentiment, recourse might be more often had to bleeding in cases where it would do undoubted good. There is not the same difficulty with the administration of antimony. It was undoubtedly abused, and we have now milder and less dangerous substitutes, but there is still reason to regard it as a valuable remedy when used with discrimination, and when its depressing effects on the heart can be avoided or allowed for. We need not now employ it as an emetic, nor in the delirium of pyrexia, since we have other and safer remedies. We need not depress and weaken the heart's action when we wish to reduce high tension, since we have remedies, such as the nitrites, which will act safely on the vasomotor mechanism itself, and so relieve the over-burdened heart. Still, there are other applications of antimony for which it is less easy to find substitutes. Thus we have seen a case of universal eczema, in which other ordinary remedies were tried in vain, yield gradually and completely to small doses of the vinum antimoniale.

Its alterative action, as Mr. Malcolm Morris and others have pointed out, is much the same as that of arsenic, to which it has a close chemical affinity. The action of arsenic in skin diseases seems to depend on the increased metabolism of the epidermis, the columnar layer of cells being more or less softened. Antimony attacks other cells besides those of the columnar layer, causing rapid softening and vacuolation. Morris finds that it causes rapid improvement in acute general eczema, when the wine is given in doses of 4 to 7 minims, especially stopping the irritation, which is such a distressing feature of these cases. Again, in erythema it both shortens the attack and alleviates the symptoms. So too in some forms of pruritus, where everything has been tried in vain, antimony in 4 minim doses of the wine affects a surprising alteration. In chronic eczema the results are less satisfactory, but in psoriasis there are instances where arsenic is is of no use, but where antimony effects an improvement. Jamieson has recently recorded a case of lichen planus cured by doses of a quarter of a grain of tartar emetic, and also cases of general exfoliative dermatitis, erythematous, and moist eczema, and other inflammatory skin troubles, treated by one-eighth grain doses with great success.

On the whole, it seems of value in those acute forms of skin disease where arsenic usually irritates. There are, in addition, some chronic forms where arsenic fails unaccountably, but which yield to antimonial treatment.

We have, then, in antimony, a remedy for cutaneous disorders of considerable importance, and which is found to be active in such small doses that no serious constitutional effects need be feared.

Something like a specific action has also been claimed for it in malarial fevers, but we are not aware of very convincing evidence on the point. In spurious croup, or the acute laryngitis of children, where the pulse is hard and full and the heart strong, antimonial wine in small repeated doses seems to have an effect which no other drug has in reducing the stridor, and doing away with the necessity for tracheotomy. No one, of course, would give it in bronchopneumonia, where the heart was feeble and the pulse soft, however much we desired a rapid expectorant; but in those strong, well-fed children who are subject to recurrent attacks of laryngeal dyspnœa with a hard pulse, the use of the wine, with a steam tent and a mild purgative, will often carry them out of the reach of danger. Indeed, our impression is that children tolerate it more easily than adults do. Certainly, in the kind of emergency we have mentioned, we have not seen any bad results from its use, though one must never forget the primary danger of antimony in its depressing action on the heart, and must watch the effects on the pulse while we administer it.

